# Amyloid β directly interacts with NLRP3 to initiate inflammasome activation: identification of an intrinsic NLRP3 ligand in a cell-free system

**DOI:** 10.1186/s41232-018-0085-6

**Published:** 2018-11-12

**Authors:** Ayaka Nakanishi, Naoe Kaneko, Hiroyuki Takeda, Tatsuya Sawasaki, Shinnosuke Morikawa, Wei Zhou, Mie Kurata, Toshihiro Yamamoto, Sheikh Mohammad Fazle Akbar, Tamotsu Zako, Junya Masumoto

**Affiliations:** 1 Department of Pathology, Ehime University Graduate School of Medicine and Proteo-Science Centre, Shitsukawa 454, Toon, Ehime 791-0295 Japan; 2 Department of Chemistry and Biology, Ehime University Graduate School of Science and Engineering, Bunkyocho 2-5, Matsuyama, Ehime 790-8577 Japan; 3 Divison of Proteo-Drug-Discovery Sciences, Ehime University Proteo-Science Center, Bunkyocho 3, Matsuyama, Ehime 790-8577 Japan; 4 Division of Cell-free Sciences, Ehime University Proteo-Science Center, Bunkyocho 3, Matsuyama, Ehime 790-8577 Japan

**Keywords:** Alzheimer’s disease, Amyloid β, NLRP3, Interleukin-1β, Inflammasome, Cell-free

## Abstract

**Background:**

Alzheimer’s disease is a neurodegenerative disease characterized by the interstitial deposition of amyloid β (Aβ) plaque, which is thought to be related to chronic neuroinflammation. Aβ is known to make fibrils via oligomers from monomers. Aβ has been reported to activate the NLRP3 inflammasome in infiltrated macrophages. NLRP3, an intracellular pattern recognition receptor, has been reported to recognize numerous pathogens and/or metabolites and form complexes with adopter protein ASC to make the inflammasome, an interleukin (IL)-1β-processing platform. Although reactive oxygen species from mitochondria have been reported to be involved in the activation of the NLRP3 inflammasome in microglial cells upon the deposition of Aβ, whether Aβ directly or indirectly activates the NLRP3 inflammasome remains unclear.

**Methods:**

We prepared monomers, oligomers, and fibrils of Aβ, which promoted the interaction between NLRP3 and each form of Aβ and analyzed the interaction between NLRP3 and ASC induced by each form of Aβ in a cell-free system with the amplified luminescent proximity homogeneous assay. We also confirmed the physiological relevance in a cell-based assay using human embryonic kidney 293T cells and human peripheral mononuclear cells.

**Results:**

Monomers, oligomers, and fibrils of Aβ were successfully prepared. Aβ oligomers and fibrils interacted with NLRP3. Aβ oligomers and fibrils induced the interaction between NLRP3 and ASC. However, Aβ monomers did not interact with NLRP3 or induce interaction between NLRP3 and ASC in the cell-free system, and IL-1β was not secreted according to the cell-based assay.

**Conclusion:**

Oligomerized Aβ originating from non-toxic Aβ monomers directly interacted with NLRP3, leading to the activation of the NLRP3 inflammasome. This may be an attractive target for the treatment of Alzheimer’s disease.

**Electronic supplementary material:**

The online version of this article (10.1186/s41232-018-0085-6) contains supplementary material, which is available to authorized users.

## Background

Alzheimer’s disease (AD) is a neurodegenerative disease, characterized by neuronal cell death accompanied by the interstitial deposition of amyloid β (Aβ) plaque and cytoplasmic phosphorylated tau protein, and neurofibrillary tangles (NFTs) [1]. Recently, evidence was found that the innate immune system was closely related to neurodegenerative diseases such as AD [2].

The inflammasome has been identified as an intracellular complex that plays an important role in innate immune responses against pathogens and toxic metabolites. NACHT, LRR, and PYD domain-containing protein 3 (NLRP3), an intracellular pattern recognition receptor, has been reported as a component of the intracellular innate immune receptor complexes with adopter protein ASC that forms inflammasome, an interleukin (IL)-1β-processing platform [2–4].

We previously developed the NLRP3 inflammasome in a cell-free system to detect intrinsic and extrinsic directly interacting ligands [5]. The NALP3 inflammasome was reported to be a sensor of phagocytosed Aβ [6]; however, Aβ did not induce an interaction with NLRP3 or apoptosis-associated speck-like protein containing a caspase recruitment domain (ASC) in the cell-free system [5].

There have been several reports that Aβ oligomerization, which is thought to play an important role in the innate immune response and cell death, is required for the pathogenesis of AD [7, 8]. It has been suggested that reactive oxygen species (ROS) are involved in the initiation of this innate response related to NLRP3 inflammasome activation, which may play a significant role in promoting the development of AD [9].

Currently, numerous ligands, including Aβ, have been reported to activate the NLRP3 inflammasome; however, none were confirmed at the molecular level in previous reports. Thus, the purpose of our study was to clarify whether Aβ oligomers directly or indirectly activate the NLRP3 inflammasome. In order to confirm direct interactions, we used the cell-free system in this study.

## Methods

### Regents

Aβ42 was purchased form the Peptide Institute (Ibaraki, Osaka, Japan). Anti-Aβ mouse monoclonal antibody (6E10) and anti-mouse immunoglobulin G (IgG) were purchased from BioLegend (San Diego, CA, USA) and R&D Systems (Minneapolis, MN, USA), respectively. Thioflavin T (ThT) was obtained from Sigma-Aldrich (St. Louis, MO, USA). Anti-FLAG monoclonal antibody M2 (F1804) was purchased from Sigma-Aldrich (St. Louis, MO, USA). Human IL-1β ELISA Set II was purchased from BD Biosciences (San Jose, CA, USA). Anti-cleaved caspase-1 rabbit monoclonal antibody (Asp297) (D57A2) (S4199) was purchased from Cell Signaling Technology (Danvers, MA, USA). MCC950 (PZ0280) was purchased from Sigma-Aldrich (St. Louis, MO). Isoliquiritigenin (I0822) was purchased from Tokyo Chemical Industry Co., Ltd. (Tokyo, Japan).

### Preparation of Aβ monomers, oligomers, and fibrils

Aβ42 stock solution (400 μM) was prepared by dissolving the lyophilized peptide in 0.1% ammonia solution. The oligomerization reaction was initiated by diluting the stock solution in 10-times diluted phosphate-buffered saline (PBS) (final concentration: 45 μM Aβ42 monomers, pH 7.4) at room temperature. For oligomer and fibril formation, Aβ42 monomers (45 μM) were incubated at room temperature for 14 h (oligomers) or at 37 °C for 48 h (fibrils).

### Native polyacrylamide gel electrophoresis and Western blotting analysis

The Aβ samples (7.5 μL) were mixed with the native-PAGE sample buffer (2.5 μL) and used for native-PAGE with an 8% gel. A NativeMark Unstained Protein Standard (Invitrogen) comprising 1236 IgM Hexamer (1236 kDa), Apoferritin band 1 (720 kDa), Apoferritin band 2 (480 kDa), and bovine serum albumin (66 kDa) was used as a molecular mass marker. Following transfer to a nitrocellulose membrane, the membrane was blocked with 5% skim milk in Tris-buffered saline including 0.05% Tween20 (TBST) for 1 h at room temperature. Then, the membrane was incubated with anti-Aβ (6E10, 1:5000) for 1 h at room temperature. After washing with TBST, it was incubated with the secondary antibody (1:10,000) for 1 h at room temperature. After washing with TBST, proteins were visualized using the ECL Plus Western Blotting Detection System (GE Healthcare, Chalfont St. Giles, UK). Luminescence was detected by the LAS4000 mini luminescent Image Analyzer (Fujifilm, Tokyo, Japan).

### Scanning electron microscopic analysis

The 10 μM Aβ samples were dropped onto a silicon wafer and allowed to air-dry. Samples were observed at an acceleration voltage of 15 kV using a field-emission scanning electron microscope (FE-SEM, JSM7001FA, JEOL: Tokyo, Japan).

### Thioflavin T spectroscopic assay

The Aβ samples (5 μM) were mixed with a ThT solution (50 μM) in PBS. Then, ThT fluorescence was measured using a microplate reader (Safire2, TECAN, Mannedorf, Switzerland) at an excitation wavelength of 450 nm and emission wavelength of 490 nm [10].

### Recombinant protein synthesis

pDONR221-NLRP3 and pDONR221-ASC were inserted into pEU-E01-GW-bls-STOP and pEU-E01-GW-STOP, respectively, for cell-free protein expression, as previously reported [5]. The entire cDNA of GFP was derived from pEU-E01-bls-GFP. The open reading frame of GFP without a stop codon was modified in a two-step polymerase chain reaction using the following primer sets:

forward primer S1-GFP_F: 5′-CCACCCACCACCACCAATGGTGAGCAAGGGCGAGG-3′ with reverse primer GFP-T1(F)_R: 5′- TCCAGCACTAGCTCCAGACTTGTACAGCTCGTCCATGC -3′. The second step was carried out using the following primer sets: attB1-S1: 5′-GGGGACAAGTTTGTACAAAAAAGCAGGCTTCCACCCACCACCACCAATG-3′ and attB2-T1: 5′-GGGGACCACTTTGTACAAGAAAGCTGGGTCTCCAGCACTAGCTCCAGA-3′. PCR products were inserted into a Gateway® pDONR™221 Vector (pDONR221) (Life Technologies, Carlsbad, CA, USA) using the Gateway® BP Clonase™ II Enzyme mix (Life Technologies, Carlsbad, CA, USA) to generate entry clones. The GFP entry clones pDONR221-GFP was inserted into pEU-E01-GW-bls-STOP using the Gateway™ LR Clonase™ II Enzyme mix (Life Technologies, Carlsbad, CA, USA) for cell-free protein expression. The constructed plasmids were used to synthesize specific proteins with a WEPRO1240 Expression Kit (Cell-free, Inc., Matsuyama, Japan), followed by Western blotting to confirm prompt protein synthesis.

### Amplified luminescent proximity homogeneous assay

Synthesized protein-protein interactions were assessed using the amplified luminescent proximity homogeneous assay (ALPHA). A total of 100 ng of each protein was added to ALPHA buffer [100 mM Tris-HCl (pH 8.0), 0.01% (*v*/*v*) Tween20], 1 mg/mL of BSA, 17 μg/mL of streptavidin-conjugated ALPHA donor beads (PerkinElmer, Waltham, MA, USA), 17 μg/mL of protein-A-conjugated ALPHA acceptor beads, and 5 μg/mL of anti-FLAG mAb M2 and incubated in a 1/2ALPHAPlate-96 shallow well (PerkinElmer, Waltham, MA, USA) at 25 °C for 24 h. The fluorescence emission signals of each well were measured using an EnSpire™ Multimode Plate Reader (PerkinElmer, Waltham, MA, USA).

The interaction between NLRP3 and Aβ was assessed by incubating 100 ng of NLRP3-FL-Btn with 5 μg/mL of anti-Aβ mAb (6E10) (BioLegend, San Diego, CA, USA), 17 μg/mL of protein-A-conjugated ALPHA acceptor beads, and 17 μg/mL of streptavidin-conjugated ALPHA donor beads for 24 h with the indicated concentrations of Aβ and incubated in a 1/2ALPHAPlate-96 shallow well (PerkinElmer, Waltham, MA, USA) at 25 °C for 24 h. The fluorescence emission signals of each well were measured using an EnSpire™ Multimode Plate Reader (PerkinElmer, Waltham, MA, USA).

### Cell-based assay using human embryonic kidney (HEK) 293T cells

Human embryonic kidney (HEK) 293T cells were maintained in DMEM (Gibco) with 10% heat-inactivated FBS, penicillin, and streptomycin. Transfection was carried out with the calcium phosphate method. A total of 1 × 10^5^ HEK 293T cells were co-transfected with the indicated amount of non-tagged expression plasmids such as pcDNA3-NLRP3, pcDNA3-ASC, pcDNA3-caspase-1, and pcDNA3-IL-1β. After hours, the transfection medium was changed to fresh medium together with Aβ monomers, oligomers, or fibrils at the indicated concentrations. IL-1β concentrations in the culture supernatants were measured 16 h after medium change using the enzyme-linked immunosorbent assay (ELISA) with specific human IL-1β antibodies (BD Biosciences, San Jose, CA, USA) according to the manufacturer’s instructions.

### Cell-based assay using human peripheral blood mononuclear cells

Human peripheral blood mononuclear cells (MNCs) were separated by Ficoll gradient centrifugation (GE Healthcare Bio-Sciences AB, Piscataway, NJ, USA) according to the manufacturer’s instructions. The cells were cultured in 24-well flat-bottom plates (BD Biosciences, San Jose, CA, USA) at a final cell density of 5 × 10^5^/mL in a volume of 1 mL of RPMI1640, including 10% FBS with Aβ oligomers, and lipopolysaccharide (LPS) or left untreated for 16 h at 37 °C in a humidified atmosphere with 5% CO_2_. Concentrations of IL-1β in the culture supernatants were measured by ELISA with specific human IL-1β antibodies (BD Biosciences, San Jose, CA, USA) according to the manufacturer’s instructions.

### Endotoxin quantification in Aβ

The levels of endotoxin contamination in Aβ monomers, oligomers, and fibrils were quantified by the Limulus amebocyte lysate assay using Endospecy, a chromogenic endotoxin-specific assay kit, according to the manufacturer’s instructions (Seikagaku-Biobusiness, Tokyo, Japan).

### Statistical analysis

Results are presented as the mean ± standard deviation of triplicate data acquisition, and the significance of differences was evaluated using the Mann-Whitney *U* test. A *p* value < 0.05 was considered significant.

## Results

### Aβ42 oligomerizations and morphological changes

**Fig. 1:**
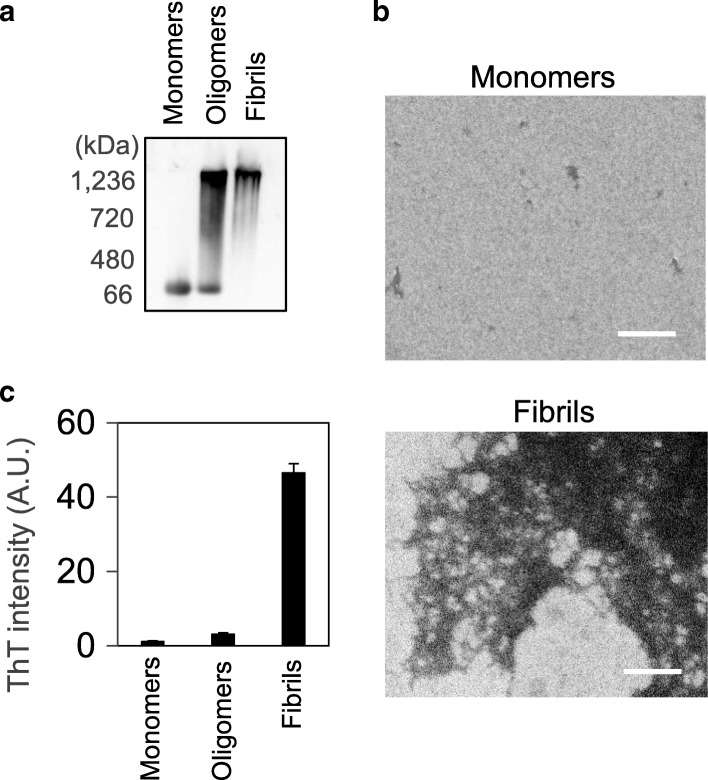
Aβ42 oligomerization and morphological changes. **a** Aβ42 oligomerization sequences. Aβ42 monomers (45 μM) were incubated under oligomerization conditions at room temperature for 0 h (monomers) or for 14 h (oligomers), or at 37 °C for 48 h (fibrils), and applied to 8% native polyacrylamide gel electrophoresis followed by Western blotting. **b** The ThT assay revealed Aβ42 assembly under oligomerization conditions at room temperature for 0 h (monomers) or 14 h (oligomers), or at 37 °C for 48 h (fibrils). **c** Morphological images of Aβ42 samples under oligomerization conditions for 0 h at room temperature (monomers) or for 48 h at 37 °C (fibrils). Scale bars are 500 nm

Aβ oligomerization reactions successfully progressed, as confirmed by native PAGE followed by Western blotting analysis, the ThT assay, and SEM analysis (Fig. 1). As shown in Fig. 1a, the formation of soluble oligomers was confirmed. The ThT assay revealed that fibrils were not included in the oligomer sample (Fig. 1b). Fibril formation was supported by the ThT assay and SEM analysis (Fig. 1b, c). Native PAGE analysis demonstrated that oligomers and monomers were not included in the fibril sample (Fig. 1a). SEM and native PAGE analyses also supported that the Aβ42 monomer sample lacked oligomers and fibrils (Fig. 1a, c).

### Aβ oligomerization induced interaction between NLRP3 and ASC in the cell-free system

**Fig. 2:**
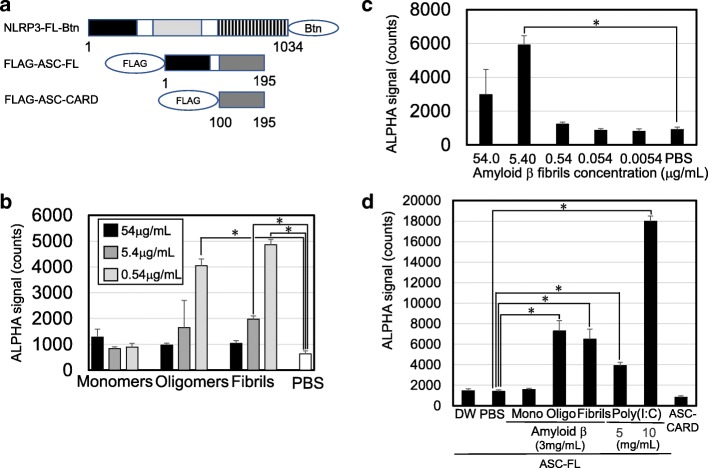
Direct interaction between Aβ and NLRP3 induced the initial event of NLRP3 inflammasome activation. **a** Schematic representations of NLRP3 and ASC. Synthetic C-terminal biotinylated full-length NLRP3 (NLRP3-FL-Btn), N-terminal FLAG-tagged ASC (FLAG-ASC-FL), and N-terminal FLAG-tagged ASC-CARD (FLAG-ASC-CARD) are indicated. The pyrin domain (PYD) is indicated by black boxes. The caspase recruitment domain (CARD) is indicated by a dark gray box. The nucleotide-binding oligomerization domain (NOD) is indicated by a light gray box. Leucine-rich repeats are indicated by a striped box. Amino acid sequence numbers are indicated under each schematic. **b** Interaction between NLRP3-FL-Btn and each form of Aβ with indicated concentrations is presented. **c** Interaction between NLRP3-FL-Btn and Aβ fibrils with indicated concentrations is presented. **d** Interaction between NLRP3-FL-Btn and FLAG-ASC-FL upon incubation with each form of Aβ with indicated concentrations. Interactions between NLRP3-FL-Btn and FLAG-ASC-FL upon incubation with poly(I:C) were positive controls. Interaction between NLRP3-FL-Btn and FLAG-ASC-CARD was the negative control. Interaction was detected by an amplified luminescent proximity homogeneous assay (ALPHA). Results are given as means ± standard deviation of triplicate data acquisition. *p value < 0.05 was considered significant

C-terminal biotinylated full-length NLRP3 (NLRP3-FL-Btn), N-terminal FLAG-tagged full-length ASC (FLAG-ASC-FL), and FLAG-tagged CARD-only ASC (FLAG-ASC-CARD) are schematically indicated in Fig. 2a. Using wheat germ cell-free system-specific expression plasmids, NLRP3-FL-Btn, FLAG-ASC-FL, and FLAG-ASC-CARD proteins were also synthesized, as described previously [5].

Next, we investigated whether Aβ monomers, oligomers, and fibrils interacted with NLRP3 under cell-free conditions with ALPHA. Direct interactions between NLRP3-FL-Btn and Aβ oligomers or Aβ fibrils were observed in a dose-dependent manner (Fig. 2b, oligomers and fibrils, respectively). However, no interaction between NLRP3-FL-Btn and Aβ monomers was observed (Fig. 2b, monomers). We further confirmed the interaction between NLRP3-FL-Btn and Aβ fibrils. The ALPHA signals of the interaction gradually increased with 5.4 μg/mL of Aβ fibrils and decreased at higher concentrations of Aβ fibrils (Fig. 2c).

Next, we tested whether Aβ induced the interaction between NLRP3-FL-Btn and FLAG-ASC-FL. Both Aβ oligomers and Aβ fibrils significantly induced the interaction between NLRP3-FL-Btn and FLAG-ASC-FL in the cell-free system (Fig. 2d, oligo and fibrils, respectively). Positive controls of poly(I:C) also induced the interaction between NLRP3-FL-Btn and FLAG-ASC-FL (Fig. 2d, poly(I:C)).

### Aβ oligomers, but not Aβ fibrils, induced IL-1β secretion from 293T cells with expression plasmids

**Fig. 3:**
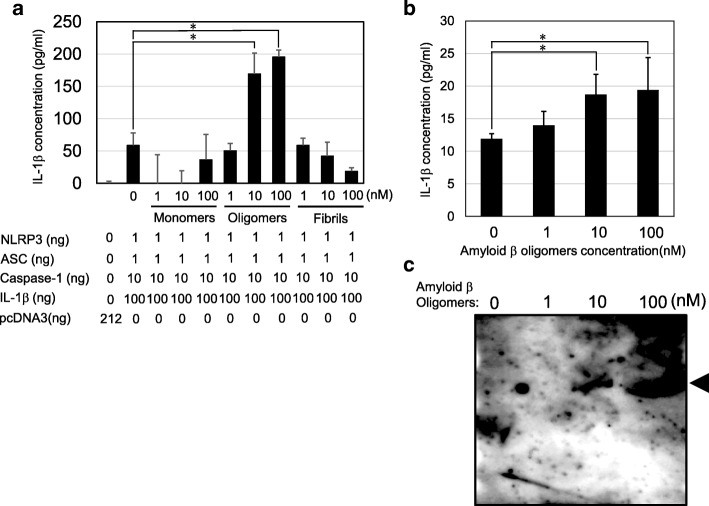
IL-1β secretion from living cells.**a** Cell-based assay using human embryonic kidney (HEK) 293T cells transfected with NLRP3, ASC, caspase-1, and IL-1β. IL-1β concentrations were evaluated by ELISA after a 16-h incubation with Aβ monomers, oligomers, or fibrils. **b** Cell-based assay using human peripheral mononuclear cells. IL-1β concentrations were evaluated by ELISA after a 16-h incubation with Aβ oligomers. **c** Caspase-1 activation in cell lysates was detected by immunoblotting corresponding to **b**. Cleaved-caspase-1 is indicated by arrowheads

The cell-based assay using 293T cell expression plasmids revealed that Aβ oligomers at 10 and 100 nM, but not monomers or fibrils, were able to activate the reconstituted-intracellular NLRP3 inflammasome leading to IL-1β secretion (Fig. 3a). In a cell-based assay using 293T cells, Aβ fibrils did not induce IL-1β secretion, different from the cell-free system (Fig. 1).

### Aβ oligomers induced IL-1β secretion and caspase-1 activation in human peripheral mononuclear cells

The physiological relevance was evaluated using human MNCs incubated with Aβ oligomers. Consistent with the cell-based assay using 293T cells, Aβ oligomers at 10 and 100 nM induced even low but significant IL-1β secretion from human MNCs and caspase-1 activation (Fig. 3b, c).

### Specificity of the cell-free assay system

To test the specificity of the cell-free assay system, the interactions between NLRP3-Btn or GFP-Btn and 4.5 μM Aβ were assessed. The ALPHA-positive ratio of the interaction between NLRP3-Btn and Aβ was much higher than that between GFP-Btn and Aβ (Additional file 1: Figure S1). Therefore, NLRP3 was able to specifically recognize Aβ in comparison with GFP (Additional file 1: Figure S1).

### Effects of known NLRP3 inhibitors on the interaction between FLAG-NLRP3-FL and Aβ in the cell-free system

We investigated the interaction between NLRP3 and Aβ oligomers and fibrils using MCC950 and isoliquiritigenin, known as NLRP3 inhibitors, in the cell-free system. The positive ALPHA signal rates for the interaction between FLAG-NLRP3-FL and Aβ oligomers or Aβ fibrils in the presence of 50 μM MCC950 in distilled water were not reduced compared with distilled water alone (*p* = 0.275) (Additional file 2: Figure S2A). Furthermore, positive ALPHA signal rates for the interaction between FLAG-NLRP3-FL and Aβ oligomers or Aβ fibrils in the presence of 50 μM isoliquiritigenin in 1% (*v*/*v*) DMSO were not reduced compared with 1% (*v*/*v*) DMSO alone (*p* = 0.275) (Additional file 2: Figure S2B).

### Endotoxin contamination in Aβ

Endotoxin contamination in Aβ monomers, oligomers, and fibrils was in an undetectable level.

## Discussion

AD has been reported as a neuroinflammatory disease linked to the pathogenesis of the hyper-secretion of IL-1β via the activation of the NLRP3 inflammasome [4]. Lysosomal damage caused by Aβ oligomers following ROS production has been reported to induce the NLRP3 inflammasome in microglial cells in AD [9, 11]. This Aβ oligomerization-initiated ROS-mediated NLRP3 inflammasome activation scenario has been accepted [12], but the possibility that Aβ directly initiates the NLRP3 inflammasome cannot be excluded.

The purpose of our study was to clarify whether Aβ oligomers directly or indirectly activate the NLRP3 inflammasome. Numerous ligands, including Aβ, have been reported to activate the NLRP3 inflammasome; however, none were confirmed at the molecular level in previous reports.

In order to identify directly stimulating endogenous ligands, we previously developed a reconstituted NLRP3 inflammasome in a cell-free system [5, 13]. In this study, we successfully prepared Aβ oligomers and Aβ fibrils (Fig. 1) and found that oligomerized Aβ directly interacted with NLRP3 and induced the interaction of NLRP3 and ASC in the cell-free system, which may be an initial event in NLRP3 inflammasome activation (Fig. 2). Although the cell-free system cannot fully reflect physiological events, this study provides evidence that helps clarify the AD pathogenesis, being consistent with a report that Aβ causes lysosomal damage, inducing ROS-mediated NLRP3 inflammasome activation [9].

Under cell-free conditions, Aβ oligomers and fibrils, but not monomers, interacted with NLRP3 (Fig. 2). As for inflammasome-related intracellular pattern recognition receptors, NLRP3 and AIM2 were reported to recognize poly(I:C) and poly(dA:dT), both of which are large-molecular-weight nucleic acid polymers, to assemble the NLRP3 and AIM2 inflammasomes [14–17]. NLRC4 was reported to recognize flagellin, which can oligomerize to form fibrils to assemble the NLRC4 inflammasome [18, 19]. Pyrin was reported to recognize actin, which can oligomerize to form fibrils to assemble the pyrin inflammasome [20, 21]. In this context, fibril formation is considered to play an important role in inflammasome formation. Indeed, fibril formation was reported to be based on conformational changes in vitro [22]. According to a study of Aβ, Aβ monomers show lower radius of gyration than the Aβ oligomers, which may be related to the binding capacity of NLRP3, resulting in NLRP3 inflammasome activation [23]. This hypothesis may explain why the Aβ monomers did not interact with NLRP3.

Unlike LPS, which is a well-known strong IL-1β inducer, Aβ oligomers induced even low-level IL-1β secretion from NLRP3 inflammasome-reconstituted-293T cells and human MNCs (Fig. 3a, b). On the other hand, Aβ fibrils were unable to induce such IL-1β secretion in the cell-based assay, being different from the cell-free assay (Fig. 3). We speculate that because Aβ forms a large complex from oligomers to fibrils, it can only reach intracellular NLRP3 in very low amounts. This may explain why little activated caspase-1 from PMCs was detected on immunoblotting (Fig. 3c).

We next examined the specificity of the cell-free system for NLRP3 versus green fluorescence protein (GFP) and found that NLRP3 was able to more specifically recognize Aβ as compared with GFP (Additional file 1: Figure S1); however, this does not rule out redundant functions of inflammasomes.

We also investigated the effects of the known NLRP3 inhibitors MCC950 and isoliquiritigenin [24, 25] on the interaction between FLAG-NLRP3-FL and Aβ in the cell-free system; however, there was no significant inhibition (Additional file 2: Figures S2A and S2B). These data suggest that there may be targets of inhibitors other than the Aβ recognition site.

## Conclusions

Aβ oligomers directly interact with NLRP3 to activate the NLRP3 inflammasome. This interaction may provide an attractive drug target to avoid neuroinflammation in AD therapy, although there are mechanisms for the NLRP3 inflammasome and AD pathology of the human brain that still require elucidation [26]. Our data may provide further understanding of these mechanisms.

## Additional files

Additional file 1: **Figure S1.** Specificity of ALPHA-positive ratio for the interaction between Aβ and NLRP3 in comparison with GFP. (PPTX 42 kb)

Additional file 2: **Figure S2.** MCC950 (A) and isoliquiritigenin (B), known NLRP3 inhibitors, did not affect the interaction between FLAG-NLRP3-FL and Aβ oligomers or fibrils in the cell-free system. (PPTX 51 kb)

## Abbreviations

AD: Alzheimer’s disease
ALPHA: Amplified luminescent proximity homogeneous assay
ASC: Apoptosis-associated speck-like protein containing a caspase recruitment domain
CARD: Caspase recruitment domain
IL: Interleukin
NLRP: NACHT, LRR, and PYD domain-containing protein
ROS: Reactive oxygen species
