# Supplementary material for: Amyloid β directly interacts with NLRP3 to initiate inflammasome activation: identification of an intrinsic NLRP3 ligand in a cell-free system
Source: Inflamm Regen. 2018 Nov 12;38:27. doi: 10.1186/s41232-018-0085-6 (PMC6231249; doi:10.1186/s41232-018-0085-6)
Supplement: Supplementary file 1 — Figure S1. Specificity of ALPHA-positive ratio for the interaction between Aβ and NLRP3 in comparison with GFP. (PPTX 42 kb) [file 41232_2018_85_MOESM1_ESM.pptx]

## Slide 1
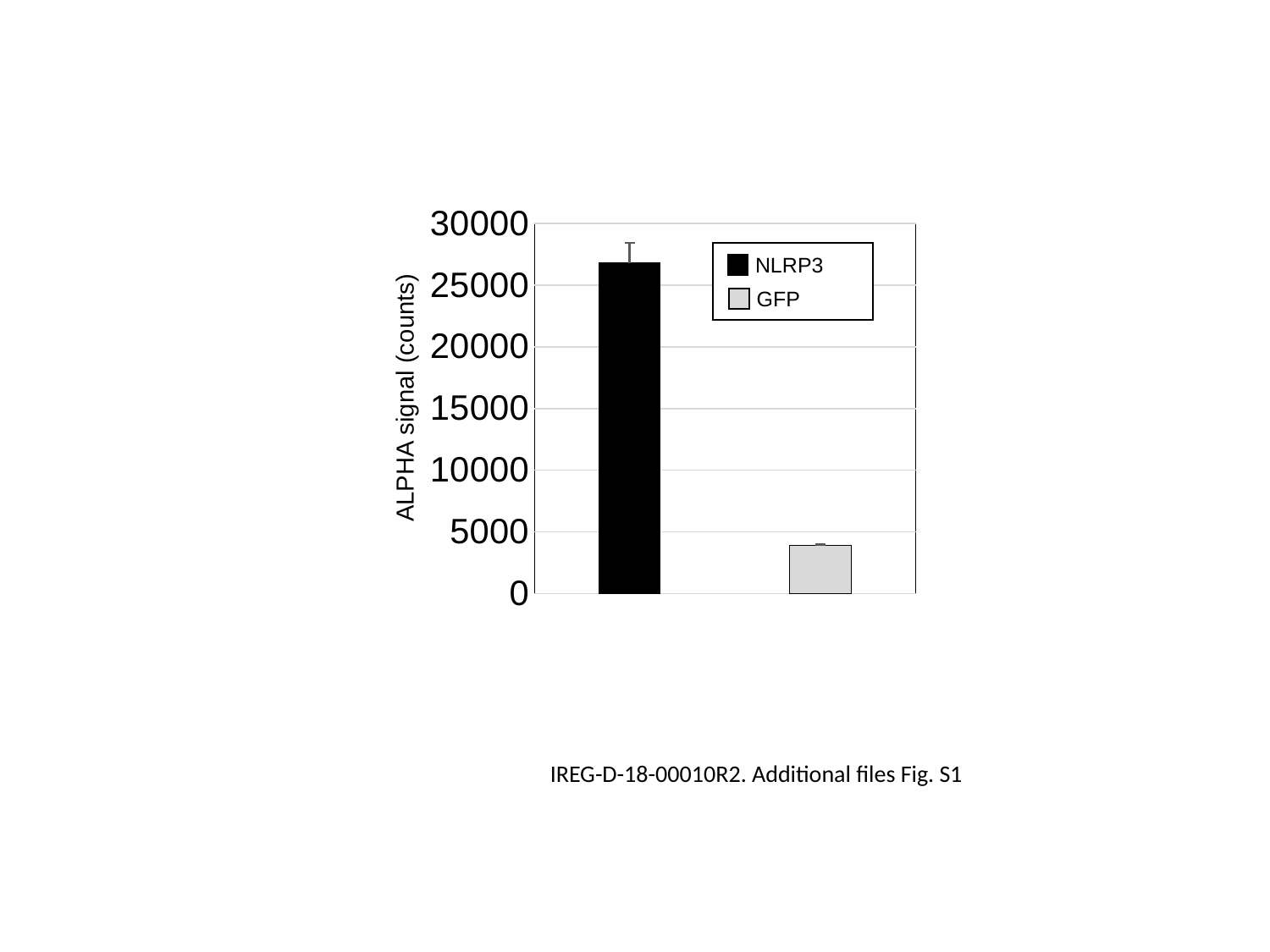

### Chart
| Category | |
|---|---|
| NLRP3-Btn | 26807.0 |
| GFP-Btn | 3924.6666666666665 |
NLRP3
GFP
ALPHA signal (counts)
IREG-D-18-00010R2. Additional files Fig. S1
