# Supplementary material for: Amyloid β directly interacts with NLRP3 to initiate inflammasome activation: identification of an intrinsic NLRP3 ligand in a cell-free system
Source: Inflamm Regen. 2018 Nov 12;38:27. doi: 10.1186/s41232-018-0085-6 (PMC6231249; doi:10.1186/s41232-018-0085-6)
Supplement: Supplementary file 2 — Figure S2. MCC950 (A) and isoliquiritigenin (B), known NLRP3 inhibitors, did not affect the interaction between FLAG-NLRP3-FL and Aβ oligomers or fibrils in the cell-free system. (PPTX 51 kb) [file 41232_2018_85_MOESM2_ESM.pptx]

## Slide 1
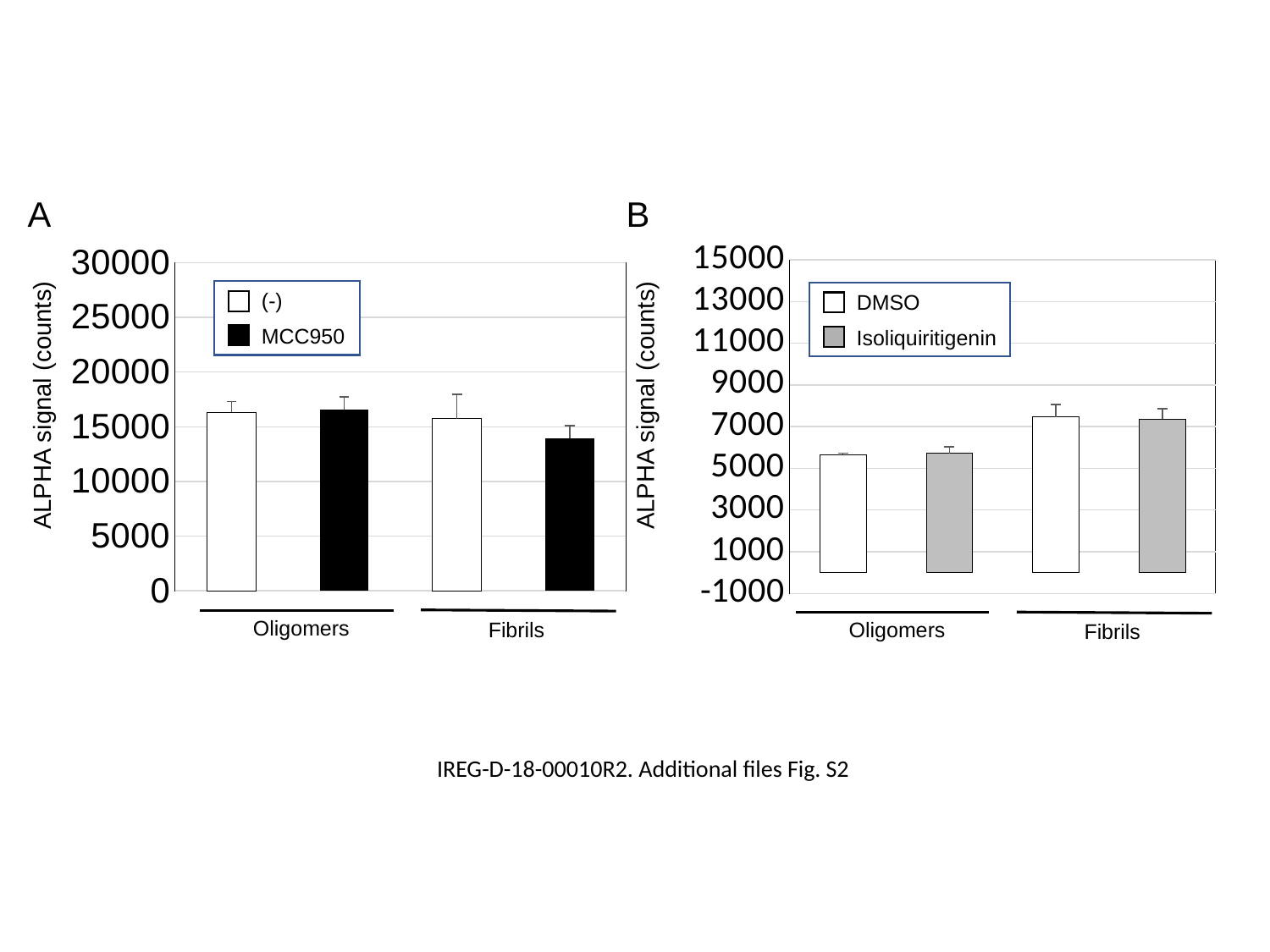

A
B
### Chart
| Category | |
|---|---|
| (-) | 5654.666666666667 |
| Isoliquiritigenin | 5715.0 |
| (-) | 7468.666666666667 |
| Isoliquiritigenin | 7365.0 |
### Chart
| Category | |
|---|---|
| (-) | 16302.333333333334 |
| MCC950 | 16585.0 |
| (-) | 15753.333333333334 |
| MCC950 | 13910.666666666666 |
(-)
MCC950
DMSO
Isoliquiritigenin
ALPHA signal (counts)
ALPHA signal (counts)
Oligomers
Fibrils
Oligomers
Fibrils
IREG-D-18-00010R2. Additional files Fig. S2
